# Gene Transfer Among Viruses Substantially Contributes to Gene Gain of Giant Viruses

**DOI:** 10.1093/molbev/msae161

**Published:** 2024-08-02

**Authors:** Junyi Wu, Lingjie Meng, Morgan Gaïa, Hiroyuki Hikida, Yusuke Okazaki, Hisashi Endo, Hiroyuki Ogata

**Affiliations:** Bioinformatics Center, Institute for Chemical Research, Kyoto University, Gokasho, Uji 611-0011, Japan; Bioinformatics Center, Institute for Chemical Research, Kyoto University, Gokasho, Uji 611-0011, Japan; Génomique Métabolique, Genoscope, Institut François Jacob, CEA, CNRS, Univ. Evry, Université Paris-Saclay, Evry F-91057, France; Research Federation for the Study of Global Ocean Systems Ecology and Evolution, FR2022/Tara GOSEE, Paris F-75016, France; Bioinformatics Center, Institute for Chemical Research, Kyoto University, Gokasho, Uji 611-0011, Japan; Bioinformatics Center, Institute for Chemical Research, Kyoto University, Gokasho, Uji 611-0011, Japan; Bioinformatics Center, Institute for Chemical Research, Kyoto University, Gokasho, Uji 611-0011, Japan; Bioinformatics Center, Institute for Chemical Research, Kyoto University, Gokasho, Uji 611-0011, Japan

**Keywords:** horizontal gene transfer among viruses, giant viruses, evolutionary rate, gene gain and loss

## Abstract

The phylum *Nucleocytoviricota* comprises a diverse group of double-stranded DNA viruses that display a wide range of gene repertoires. Although these gene repertoires determine the characteristics of individual viruses, the evolutionary processes that have shaped the gene repertoires of extant viruses since their common ancestor are poorly characterized. In this study, we aimed to address this gap in knowledge by using amalgamated likelihood estimation, a probabilistic tree reconciliation method that infers evolutionary scenarios by distinguishing origination, gene duplications, virus-to-virus horizontal gene transfer (vHGT), and gene losses. We analyzed over 4,700 gene families from 195 genomes spanning all known viral orders. The evolutionary reconstruction suggests a history of extensive gene gains and losses during the evolution of these viruses, notably with vHGT contributing to gene gains at a comparable level to duplications and originations. The vHGT frequently occurred between phylogenetically closely related viruses, as well as between distantly related viruses with an overlapping host range. We observed a pattern of massive gene duplications that followed vHGTs for gene families that was potentially related to host range control and virus–host arms race. These results suggest that vHGT represents a previously overlooked, yet important, evolutionary force that integrates the evolutionary paths of multiple viruses and affects shaping of *Nucleocytoviricota* virus gene repertoires.

## Introduction

The viral phylum *Nucleocytoviricota* (Koonin et al. 2020) encompasses diverse large and giant double-stranded DNA viruses that infect a wide spectrum of eukaryotes, from protists to mammals, with some members possessing as many as thousands of genes (Sun et al. 2020; Schulz et al. 2022). By encoding numerous unknown or uncharacterized genes, nucleocytoviruses challenge the conventional “Gene Pickpocket” hypothesis (Moreira and López-García 2005), which postulates that viruses largely acquire new genes from their host cellular organisms through horizontal gene transfer (HGT). Although many phylogenetic gene trees support HGT from cellular organisms (Monier et al. 2009, 2017; Rozenberg et al. 2020), up to 70% of the nucleocytovirus genes are unique to the phylum and lack detectable homologs in the cellular world. In the “Gene Pickpocket” hypothesis, the lack of cellular homologs of these viral genes has been explained by high mutation rates or relaxed functional constraints that erased trackable ancestral signals (Moreira and López-García 2009; Moreira and López-García 2015). However, the genes present only in nucleocytoviruses show regular features in their sequence evolution without markedly high substitution rates (Ogata and Claverie 2007), decreasing the likelihood of a recent cellular origin.

In addition to HGT from cellular organisms, gene duplication has also been suggested as a crucial mechanism driving gene gain in nucleocytovirus evolution. Suhre (2005) found that one-third of the genes in a mimivirus have at least one paralog in its genome. Also, a continuous passage experiment of a modified vaccinia virus with reduced fitness in HeLa cells resulted in gene duplication, thereby providing immediate fitness advantages (Elde et al. 2012). These examples underscore the importance of gene duplication in the expansion of the gene repertoire of nucleocytoviruses during their evolution. Gene gain can also possibly occur through de novo creation. Previous studies have shown such mechanisms for cellular organisms, with the progressive constitution of proto-genes to protein-encoding genes through successive mutations and selection (Carvunis et al. 2012; Reinhardt et al. 2013). The same mechanisms may also occur for viral genomes during their replication and could partially explain the high proportion of unique genes in nucleocytoviruses, as proposed for pandoraviruses (Legendre et al. 2019).

Another possible evolutionary mechanism for gene gain in nucleocytoviruses is virus-to-virus HGT (vHGT). vHGT is considered as one of the main driving forces in the evolution of phages (Botstein 1980; Díaz et al. 1990; Mavrich and Hatfull 2017). However, only a few cases have been reported on the existence of vHGT in nucleocytoviruses so far. For instance, the phyletic patterns of gene presence and absence suggested potential vHGT events between the mimivirus and marseillevirus lineages through the coinfection of the two viruses in the same ameba cell (Boyer et al. 2009). Other evidence emerged from the observation of inconsistencies between gene trees and the species tree; virmyosin (Kijima et al. 2021) and viractin (Da Cunha et al. 2022) genes were transferred among viruses of the *Imitervirales* order and a gene of unknown function was transferred between pandoraviruses and mollivirus (Legendre et al. 2015). These cases suggest the importance of vHGT in these viruses. However, previous systematic evolutionary reconstructions of nucleocytovirus genomes used methods that do not distinguish different mechanisms of gene gains (Maruyama and Ueki 2016; Koonin and Yutin 2018), which hampered the investigation of the impact of vHGT on nucleocytovirus evolution compared with other gene gain mechanisms.

To investigate how the modern nucleocytovirus gene repertoires were shaped from those of their ancestors, we employed the amalgamated likelihood estimation (ALE; Szöllősi et al. 2013) method, a probabilistic tree reconciliation approach to detect three classes of gene gain (gene duplication, HGT, and origination) and gene loss. We generated a robust viral tree (“species tree”) for 195 reference nucleocytoviruses (mostly cultured ones) and performed tree reconciliations of over 4,700 gene families to infer evolutionary processes. In this framework, an inferred HGT is likely to be a vHGT. An origination event corresponds to the first appearance of the gene family in the viral tree, which can be attributable to an HGT from outside the viral tree (i.e. cellular organisms or other viruses not represented in our dataset), de novo gene creation, or vertical inheritance from an ancestral virus not represented in the viral tree (in the case of the origination at the root of the viral tree). Our results delineated massive and approximately equal levels of gene gain and loss events during the evolution of the gene repertoires of nucleocytoviruses. A breakdown of gene gains by mechanisms further revealed comparable levels of contribution among vHGT, gene duplications, and originations.

## Results

### Robust Viral Tree for *Nucleocytoviricota*

**Fig. 1.**
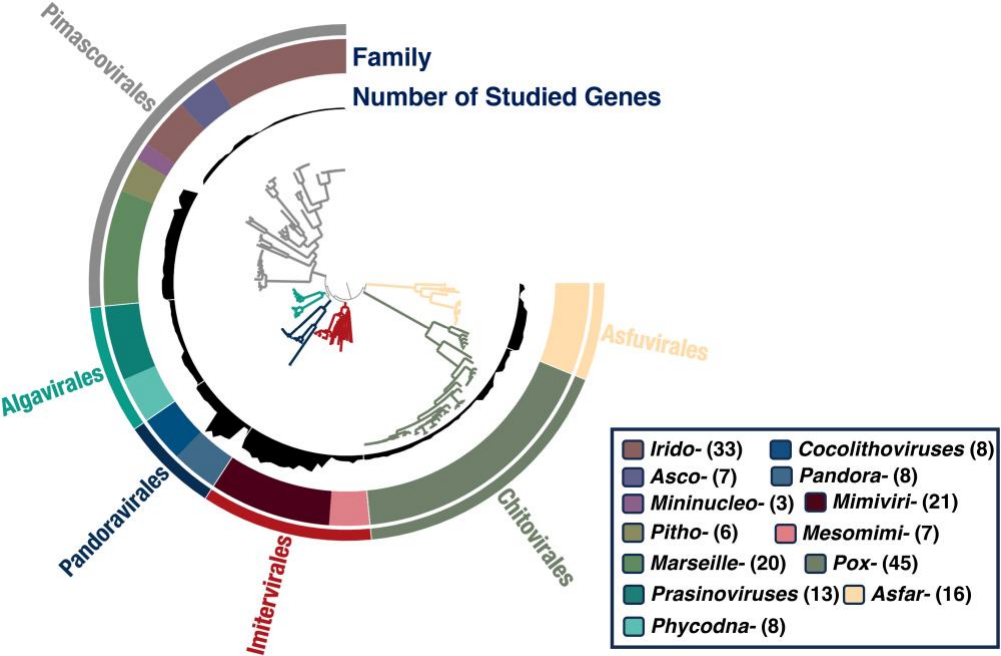
Viral tree of *Nucleocytoviricota*. The phylogenetic tree is based on concatenated seven marker protein sequences and constructed using IQ-TREE with the Q.pfam + F + I + I + R8 + C60 model. The outer layer designates the viral orders, the middle layer represents viral families, and the inner layer indicates the number of genes (ranging from 11 to 1,207) that were successfully used for tree reconciliation analysis. The number in parentheses in the legend panel indicates the number of viruses in each family, and “-” is the abbreviation of “*viridae.*”

A prerequisite for tree reconciliation by ALE for evolutionary inference is a robust phylogenetic tree for viruses that reflects their speciation events. By using seven concatenated marker genes that are considered important for viral replication and display consistent phylogenetic signals (Aylward et al. 2021), we reconstructed a phylogenetic tree of nucleocytoviruses (hereafter called the viral tree) based on genomic data from the RefSeq database and other sources. After applying an iterative process to remove, by manual inspection, viruses that contribute to unstable branches, such as medusavirus and *Heterosigma akashiwo* virus, we obtained the final viral tree (Fig. 1). In this viral tree, all branches were supported (UFboot > 95% or SH-aLRT > 80%) except one deep branch (i.e. the position of the recently proposed viral order *Pandoravirales* with UFboot = 88.8, SH-alRT = 69; Aylward et al. 2021). The viral tree contained 195 nucleocytoviruses that span six viral orders: the *Chitovirales* and the *Asfuvirales* orders of the *Pokkesviricetes* class and the *Imitervirales*, *Algavirales*, *Pandoravirales*, and *Pimascovirales* orders of the *Megaviricetes* class. The viral tree was rooted between the two classes, following the International Committee on Taxonomy of Viruses taxonomy (Koonin et al. 2019) (Fig. 1). This viral tree was then used as the reference for tree reconciliation of individual gene trees for different gene families (orthogroups [OGs]), which allows for the inference of different evolutionary events (gene gains and losses).

### Gene Trees for Reconciliation

Predicted genes in the 195 viral genomes were grouped into 8,876 OGs, excluding singletons. Of these, a total of 4,782 OGs met the requirements for confident ALE reconciliations (see the section Gene Tree and Viral Tree Reconciliation in Materials and Methods), and evolutionary scenarios involving gene gain and loss were inferred for them (supplementary table S1, Supplementary Material online, for example, see Materials and Methods). These OGs cover an average of 74.3% of genes from individual viral genomes (supplementary fig. S1, Supplementary Material online).

### The Compensation of Massive Gene Loss by Gene Gain

**Fig. 2.**
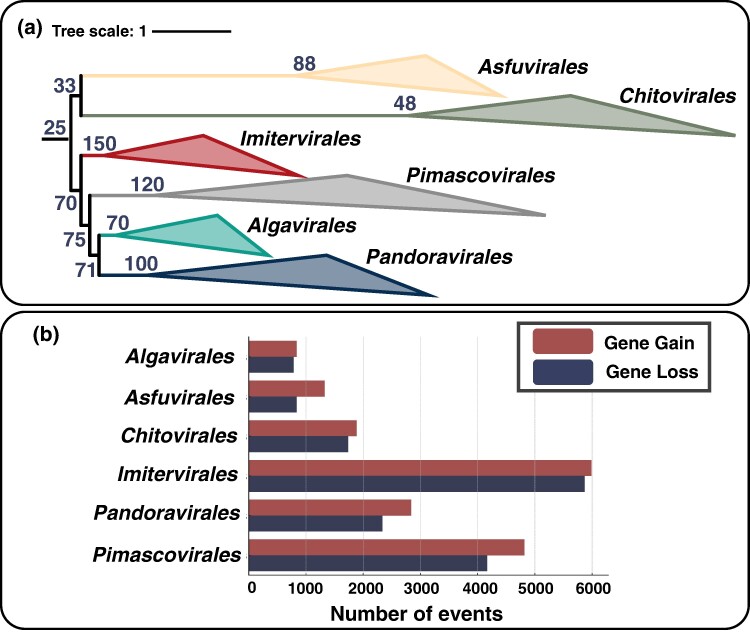
Evolutionary inference of gene gain and loss events for *Nucleocytoviricota*. a) The phylogenetic tree with the number of genes inferred to be present at the ancestral nodes. Leaves are collapsed at the level of viral orders. The unit of the tree scale is the number of substitutions per site. b) The number of gene gain and loss events for individual viral orders.

Reconciliation of 4,782 gene trees with the reference viral tree allowed us to infer 17,826 gene gain and 15,785 gene loss events, along with a conservative estimate of the number of genes at ancestral nodes (Fig. 2a). The number of genes in the ancestral genomes of viral orders ranged from 48 for *Chitovirales* to 150 for *Imitervirales*. The ancestor of the class *Pokkeviricetes* was inferred to encode at least 33 genes, while the ancestor of *Megaviricetes* had 70 genes. The ancestral genome at the root of *Nucleocytoviricota* was inferred to encode at least 25 genes. The proportion of gene loss events (average 46.96%) was comparable to the proportion of gene gain events (average 53.04%) over the course of evolution irrespective of viral orders (Fig. 2b). For example, the extant mimivirus encodes 805 genes considered for the evolutionary reconciliation. Over the course of the evolution from the root of *Nucleocytoviricota* to the extant mimivirus, it was inferred that the virus acquired 1,553 genes and lost 683 genes (see supplementary table S1, Supplementary Material online for other examples).

### vHGT Contributes to Gene Gains at a Comparable Level to Two Other Mechanisms

**Fig. 3.**
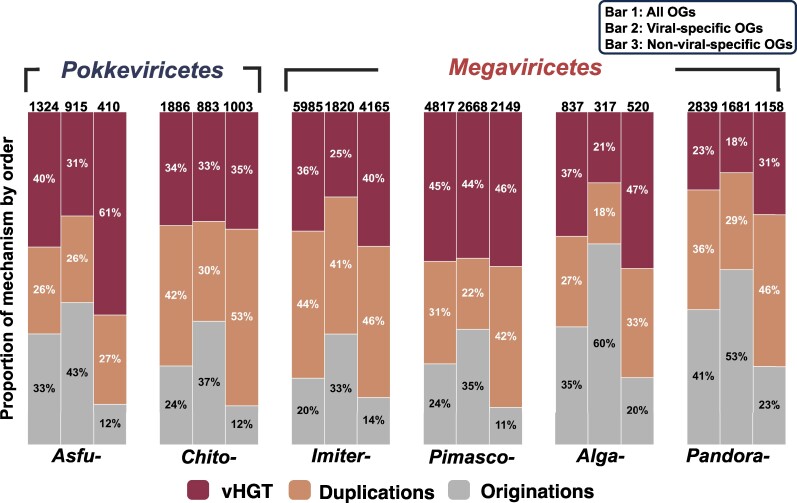
Contributions of different gene gain mechanisms in different OG sets. This bar plot depicts the contribution of each gene gain mechanism (vHGT, duplications, and origination) in all OGs, viral-specific OGs, and nonviral-specific OGs with the total number of gene gains indicated above each bar.

The ALE method can help distinguish three mechanisms of gene gain: origination, gene duplication, and highly probable vHGT. Our evolutionary reconstruction revealed that the contributions of these three mechanisms to the genome evolution were comparable (Fig. 3). The contribution of gene duplication to gene gain varied from 26% (*Asfuvirales*) to 44% (*Imitervirales*), while the contribution of origination accounted for from 20% (*Imitervirales*) to 41% (*Pandoravirales*). The contribution of vHGT varied from 23% (*Pandoravirales*) to 45% (*Pimascovirales*).

To assess the methodological dependence of these results, we tested another category of method, a parsimony-based approach implemented in Rapid Analysis of Gene family Evolution using Reconciliation-DTL (Ranger-DTL), to infer gene duplication, vHGT, and gene loss events. Ranger-DTL requires the setting of cost parameters for duplication (D), vHGT (T), and loss (L) events for evolutionary inference. In addition to the set of costs suggested by the ALE results (D/T/L = 3:3:1), we used various parameter sets to infer the evolutionary events (supplementary table S2, Supplementary Material online). Overall, the evolutionary inference using Ranger-DTL indicated a higher level of vHGT than gene duplication, except when the cost of vHGT was set to at least two times higher than the cost of duplication. This suggests that our ALE-based inference is unlikely to overestimate the vHGT events. We also performed an additional ALE-based inference on the gene families (*n* = 572) that potentially contain introns, as our gene call did not consider the presence of introns. The contributions of gene duplication (39.7%) and vHGT (47.7%) to gene gains for these gene families were comparable after considering gene fragmentation potentially caused by the presence of introns (supplementary table S3, Supplementary Material online).

As another potential artifact, the above approach could artificially recognize the parallel acquisitions of a cellular gene (or a gene homologous to a cellular gene) by multiple nucleocytoviruses as vHGT. To exclude this possibility when assessing the contribution of vHGT, we focused on a subset of the 4,782 OGs that were absent in cellular organisms and thus specific to viruses. Because parallel acquisitions by viruses from cellular organisms are unlikely for those OGs, we labeled these OGs as viral-specific OGs. We observed 3,340 (70%) viral-specific OGs. The contribution of vHGT for this subset varied from 18% (*Pandoravirales*) to 44% (*Pimascovirales*), and it was substantially reduced from the result for the entire OG set for *Asfuvirales* (from 40% to 31%), *Imitervirales* (from 36% to 25%), and *Algavirales* (from 37% to 21%). The level of gene duplication for the viral-specific OGs ranged from 18% to 41%. These results suggest that parallel acquisition of cellular genes (Rigou et al. 2022) may not be negligible in the analysis of the all OG set. However, the comparable frequency of vHGT and gene duplication events were supported by both the entire and viral-specific OG datasets. One example of the visualization of reconciliation for an OG is shown in supplementary fig. S2, Supplementary Material online.

Finally, we assessed the robustness of the ALE inference results in relation to the certainty of gene trees represented by the tree certainty all (TCA) measure (Kobert et al. 2016). TCA can range from 0 to 1, with 1 indicating no conflict in bootstrap trees and 0 indicating complete conflict. This analysis was conducted on all OGs (supplementary fig. S3a, Supplementary Material online). The result again indicated comparable levels of vHGT (34%) and gene duplication (44%) for the set of gene trees with high TCA values (>0.66) (supplementary fig. S3b, Supplementary Material online).

### Higher vHGT Frequency Between Closely Related Viruses and Between Viruses Sharing the Same or Similar Hosts

**Fig. 4.**
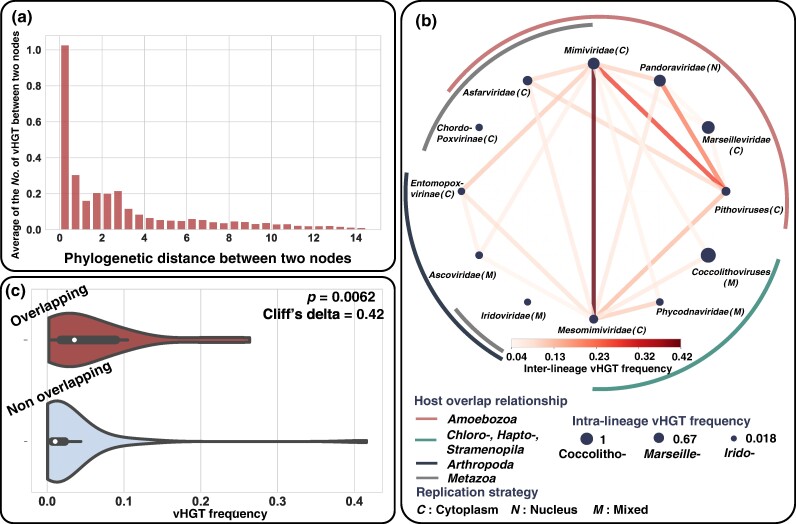
Frequency of vHGT between viruses of different phylogenetic distances and host types. a) The bar plot shows the average number of vHGT along the phylogenetic distance between the donor and recipient viruses. Note that the inferred number of vHGT for a specific branch can be below 1 (such as 0.1 or 0.5) because of the probabilistic assignment of a single event to different evolutionary processes, such as vHGT versus duplication, by ALE. b) The network represents the frequency of inter-lineage vHGT, while the filled circles represent the frequency of intra-lineage vHGT. c) The violin plot shows the frequency of inter-lineage vHGT between viral lineages with overlapping host types and those without overlapping host types. Statistical analysis was performed using the Mann–Whitney *U* test, and the effect size (Cliff's delta) is indicated. In b) and c), the direction of donor and recipient was ignored and the frequency of vHGT was summed, respectively.

We next investigated the evolutionary distances and host types between the donor and recipient viruses for the detected vHGTs to better understand the mechanisms underlying this phenomenon. The number of vHGTs between two viruses (including internal nodes) clearly showed an elevated frequency for closely related viruses (Fig. 4a), indicating prominent intra-lineage vHGT events over inter-lineage cases. For inter-lineage vHGTs (mostly at the family level, with the exception of coccolithoviruses and pithoviruses), the frequency of vHGT varied depending on the lineage (Fig. 4b). To further explore the inter-lineage vHGT, we considered that two lineages share overlapping host types if the members of both lineages can infect hosts of the same phylum. We then identified that viruses from different lineages/families that share overlapping host types display a significantly higher frequency of vHGT than those without overlapping host types (Mann–Whitney *U* test, *P* = 0.0062; Fig. 4b and c). Apart from this general trend, the *Mimiviridae* and *Mesomimiviridae* families and pithoviruses displayed a high frequency of vHGT with viral lineages of both overlapping and nonoverlapping host types, suggesting their high potential for genetic exchanges. Conversely, viruses of the *Marseilleviridae*, *Iridoviridae*, and *Chordopoxvirinae* families exhibited relatively low frequencies of inter-lineage vHGT, even with lineages that have similar host types. Furthermore, we did not observe any clear correlation between the frequency of inter-lineage vHGT and viral replication location. For example, among the ameba-infecting viruses analyzed in this study, pandoraviruses uniquely replicate in the host cell nucleus. These viruses exhibit relatively high frequencies of vHGT with other ameba-infecting viruses that replicate in the cytoplasm. In contrast, marseilleviruses that replicate in the host cell cytoplasm showed low inter-lineage vHGT frequencies, even with ameba-infecting viruses that replicate in the cytoplasm, such as mimiviruses, pithoviruses, and asfarviruses. These findings collectively suggest the importance of coinfection for enabling gene transfer, while the influence of replication location on vHGT frequency remains unclear.

### Propensity of Gene Families for vHGT

**Fig. 5.**
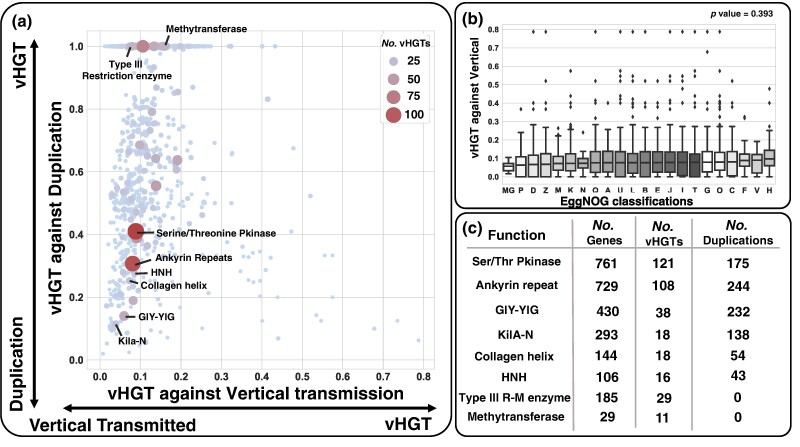
vHGT propensity against vertical evolution and gene duplication. a) vHGT propensity against vertical evolution (*x* axis) and gene duplication (*y* axis). b) vHGT propensity against vertical evolution for genes of different functional categories. Statistical analysis was performed using the Kruskal–Wallis test. Marker genes used for the viral tree reconstruction (MG), inorganic ion transport and metabolism (P), chromosome partitioning (D), cytoskeleton (Z), envelope biogenesis (M), transcription (K), cell motility (N), secondary metabolites biosynthesis (Q), RNA processing and modification (A), intracellular trafficking (U), replication, recombination, and repair (L), chromatin structure and dynamics (B), amino acid transport and metabolism (E), translation (J), lipid transport and metabolism (I), signal transduction mechanisms (T), carbohydrate transport and metabolism (G), posttranslational modification (O), energy production and conversion (C), nucleotide transport and metabolism (F), defense mechanisms (V), and coenzyme transport and metabolism (H). c) Examples of gene families with a large number of vHGTs and different levels of gene duplications ordered by the number of vHGTs and number of duplications.

We then assessed the propensity of gene families for vHGT using the relative frequencies of vHGT, vertical transmission, and gene duplication. The vHGT propensity relative to vertical transmission was defined as the frequency of vHGT divided by the sum of the frequencies of vHGT and vertical transmission for a given gene family. This is represented as follows: $P_{\mathrm{vHGT}}^{\mathrm{VT}}=f_{\mathrm{vHGT}}/[\;f_{\mathrm{vHGT}}+f_{\mathrm{VT}}]$; a value of 0.5 indicates an equal level of the two mechanisms. The vHGT propensity relative to gene duplication was similarly defined as follows: $P_{\mathrm{vHGT}}^{D}=f_{\mathrm{vHGT}}/[\;f_{\mathrm{vHGT}}+f_{D}]$. Our analysis, which encompassed 2,772 gene families (57.92% of the studied gene families) with detected vHGT events, revealed that $P_{\mathrm{vHGT}}^{\mathrm{VT}}$ was low (median = 0.086, average = 0.100, SD = 0.066). The gene families for nucleotide transport and metabolism (F), defense mechanisms (V), and coenzyme transport and metabolism (H) showed the highest $P_{\mathrm{vHGT}}^{\mathrm{VT}}$ values, although $P_{\mathrm{vHGT}}^{\mathrm{VT}}$ did not show statistically significant differences among the different functional categories (*P* = 0.389; Fig. 5b). Compared with $P_{\mathrm{vHGT}}^{\mathrm{VT}}$, $P_{\mathrm{vHGT}}^{D}$ exhibited a wide range of values (median = 1.0, average = 0.87, SD = 0.24), reflecting the varying levels of gene duplications across gene families. Additionally, it was not related to the frequency of vHGTs. Selected examples of high vHGT (*n* > 10) with functional annotations are shown in Fig. 5c (for the full list of gene families with the number of vHGT > 10, see Supplementary Data, Supplementary Material online). These gene families exhibited different levels of gene duplication. Among the families with gene duplications, we observed cases of multiple gene duplication events following vHGTs (supplementary figs. S5 to S10, Supplementary Material online).

### Conservative Estimates of the Rates of Gene Repertoire Changes

**Fig. 6.**
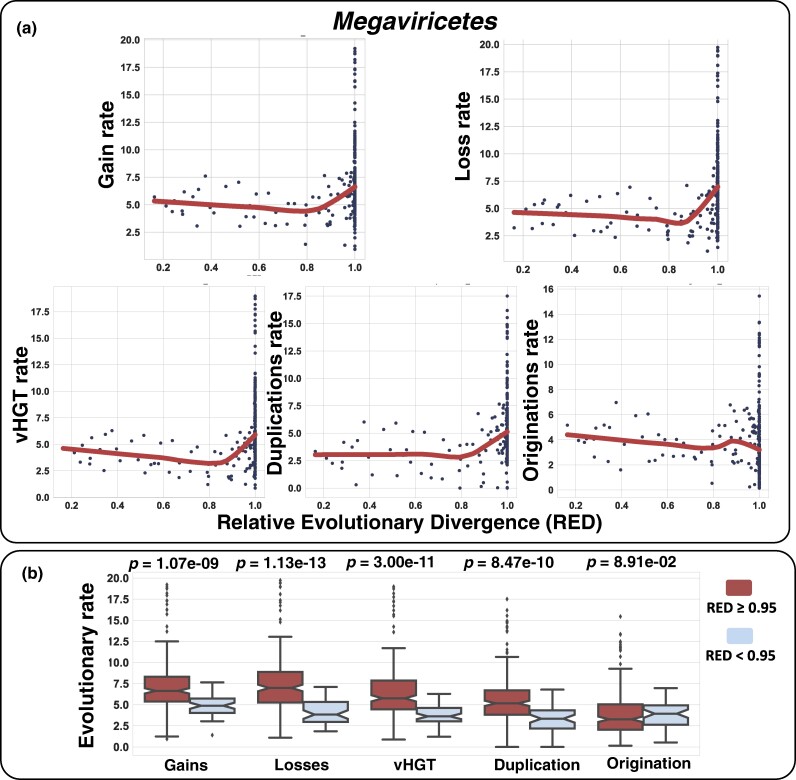
Rates of different evolutionary events along the divergence of *Megaviricetes.* a) The evolutionary rates for different evolutionary events are plotted against the divergence measured by RED. The thick line represents the local regression with LOESS (with parameter “frac” = 0.9). b) Boxplot provides a comparison of evolutionary rates between recent (RED ≥ 0.95) and earlier periods (RED < 0.95). *P*-values by the Mann–Whitney *U* test are shown above the graph.

To investigate the timing of evolutionary events, we calculated evolutionary rates as the number of evolutionary events normalized to the length of the branch in which the events occurred. We first calculated relative evolutionary divergence (RED) (Parks et al. 2018) values for the nodes of the viral tree. The RED value serves as a measure of divergence time, with “0” corresponding to the root of the tree and “1” corresponding to the leaf (extant viruses). When the rates of gene gain and loss were plotted against the RED value, there was a clear acceleration in the rates in the recent past (high rates near RED = 1) (Fig. 6, supplementary fig. S4, Supplementary Material online). Evolutionary rates (except originations) were significantly higher for the recent past (RED ≥ 0.95) than for the period before (RED < 0.95) for gene gain, loss, duplication, and vHGT (Mann–Whitney *U* test, *P* < 0.001; Fig. 6 and supplementary fig. S5, Supplementary Material online). The evolutionary inference of moumouvirus genomes, for example, depicts the high rates of recent evolution. Three moumouviruses had diverged in recent past (RED = 0.98), and their mean average nucleotide identity (ANI) was 84.7%. In the period from RED = 0.98 to 1, they gained and lost a lot of genes: the average gene gain was 63 and the average gene loss was 162.

## Discussion

In the present study, we demonstrated that vHGT contributes to nucleocytovirus gene gains at a comparable level to gene duplication and origination (Fig. 3), regardless of whether all genes or only viral-specific genes were analyzed (Fig. 3). This suggests that nucleocytoviruses can acquire genes from three principal genetic pools: genomes of their own, of host organisms, and of other related viruses. Their own genomic material can support the generation of new genes by gene duplication or de novo creation. The genetic pool of cellular organisms can also be accessed through HGT (Irwin et al. 2022), potentially broadening their functional repertoires. Lastly, vHGT allows nucleocytoviruses to access the gene pool of other nucleocytoviruses. By bridging the gene pools of viruses, vHGT allows for interplay between the evolutionary paths of different viruses and helps these viruses to collectively maintain a large and unique genetic pool.

We found that vHGT occurred more frequently between phylogenetically closely related viruses than between distantly related viruses (Fig. 4a), which is consistent with observations in bacteria and archaea (Andam and Gogarten 2011). The occurrence of inter-lineage vHGT was enriched for lineages with overlapping host types, although less frequent than the vHGT between closely related viruses (Fig. 4b and c). These results suggest the existence of “highways” for vHGT between viruses infecting the same host and can be explained by (i) “opportunities” for gene exchanges and (ii) the “usability” of transferred genes (as the recipient virus may be under similar selection pressure as the donor). No strong relationship was observed between the frequency of vHGT and functional categories of genes (Fig. 5b). However, the genes with a history of many vHGT events included notable functions, as discussed below.

Although inter-lineage vHGT was not as prominent as intra-lineage vHGTs in terms of frequency, such events may have been significantly beneficial (Fig. 4b). In the phylum *Nucleocytoviricota*, viruses exhibit a broad and phylogenetically heterogeneous host tropism (Sun et al. 2020), suggesting frequent host switching during their evolution. We observed vHGTs for a gene family potentially related to viral host range. The KilA-N domain-containing protein family is related to the host tropism of poxviruses (Bratke et al. 2013), and a substantial number of vHGT events were observed (*n* = 18) for this family. From its most likely origination position located in the ancestor of *Entomopoxvirinae* (supplementary fig. S5, Supplementary Material online), the KilA-N gene was likely transferred from *Entomopoxvirinae* to *Mimiviridae*. *Mimiviridae* members may have benefited from this putative vHGT by increasing their ability to infect a broad range of hosts. Intriguingly, *Mimiviridae* viruses exhibit a high vHGT frequency, with both lineages sharing overlapping and nonoverlapping host types (e.g. the vHGT frequency between *Mimiviridae* and *Entomopoxvirinae* [nonoverlapping host type] is higher than that between *Ascoviridae* and *Entomopoxvirinae* [overlapping host type]). Such a pattern suggests a wide range of unknown host types for the *Mimiviridae* members.

Our evolutionary reconstruction of the KilA-N domain-containing protein family further revealed an intriguing pattern of evolution, characterized by numerous gene duplications following vHGT events (supplementary fig. S5, Supplementary Material online). Such a pattern of evolution suggests strong benefit from having multiple copies of a gene, similar to the case observed in the modified vaccinia virus (Elde et al. 2012). This pattern of evolution occurred multiple times in the history of the KilA-N domain-containing protein family (the *Mimiviridae* clade and the *Entomopoxvirinae* clade; supplementary fig. S5, Supplementary Material online). Similar cases of vHGT followed by massive gene duplications were also observed for other genes related to host range (e.g. ankyrin repeats; supplementary fig. S6, Supplementary Material online; Bratke et al. 2013) and virus–host interactions (e.g. collagen triple helix repeat [supplementary fig. S7, Supplementary Material online, Mrázek and Karlin 2007] and serine/threonine protein kinase [supplementary fig. S8, Supplementary Material online, Jacob et al. 2011]). We noted that these two families (ankyrin repeats and collagen triple helix repeat) are composed of repeated sequences, for which sequence alignment and tree reconstruction are generally difficult. Although they exhibited relatively high TCA measures (supplementary fig. S3c, Supplementary Material online), which usually ensure a high reliability of evolutionary inference, we acknowledge that further in-depth validation may be needed for the inferred evolutionary scenarios for such protein families.

In contrast to the above cases, certain gene families experienced a large number of vHGTs (*n* > 10) without subsequent gene duplications. For example, the methyltransferase gene family and type III restriction enzyme genes experienced substantial numbers of vHGT events (*n* = 11 and *n* = 29, respectively) with no clear evidence of gene duplication. The transfer of both gene families, likely constituting the restriction modification systems, may provide benefits related to the virus–host arms race (Rao et al. 2014; Jeudy et al. 2020).

The highways of vHGT also facilitate the spread of selfish genetic elements. GIY-YIG domain-containing proteins (supplementary fig. S9, Supplementary Material online) and HNH domain-containing proteins (supplementary fig. S10, Supplementary Material online) are endonucleases often used by selfish genetic elements, such as introns and inteins, for the purpose of integration into genomic DNA (Dunin-Horkawicz et al. 2006). These elements are sometimes recruited by viral genes, for example, for DNA repair functions (Ogata et al. 2011). The GIY-YIG domain-containing gene family experienced 38 vHGT events and 232 gene duplication events, while the HNH domain-containing protein family experienced 16 vHGT events and 43 gene duplication events (Fig. 5c). Our inference suggests that there was a vHGT between distantly related lineages for the GIY-YIG family (supplementary fig. S9, Supplementary Material online), with the common ancestor of *Entomopoxvirinae* being the possible donor and the common ancestor of *Ascoviridae* and *Iridoviridae* being the possible recipient. These ancestral donor and recipient viruses could have possibly shared the same host. After the transfer, the selfish elements likely widely colonized in these insect-infecting viruses by gene duplication.

The ALE tree reconciliation method can systematically and quantitatively identify evolutionary events. However, some limitations still exist. Previous work (Koonin and Yutin 2010; Maruyama and Ueki 2016) and the current study identified only a small portion of genes as inherited from ancestral viruses (Fig. 2). However, the inferred number of genes in the ancestral nucleocytoviruses is likely a conservative estimate because of two limitations when inferring evolutionary events using genomic data. Firstly, the inference is affected by the sampling of extant viruses. The virus genomes used in our study do not fully represent the actual diversity of viruses in nature. For example, the highly diverse nucleocytovirus genomes recovered from metagenomes (Moniruzzaman et al. 2020; Schulz et al. 2020; Gaïa et al. 2023) were not considered in this study because of the incompleteness of their gene repertoires. The ALE method likely missed the evolutionary events involving unsampled viruses, notably including many extinct viruses in deep branches. Secondly, genes that have been lost across all studied viruses cannot be incorporated into the evolutionary inference process. Consequently, the number of genes inferred to have been present in ancestral nucleocytoviruses (Fig. 2) does not necessarily represent their gene content. For example, the last common ancestor of *Imitervirales* was inferred to have possessed 150 genes, which represent the genes successfully inherited by some of the extant *Imitervirales* members analyzed in this study. Therefore, our analysis provides conservative estimates for gene gain and loss events, especially in deeper branches.

We observed an apparent acceleration of evolutionary rates as depicted by “J-shape” curve (Fig. 6a and supplementary fig. S4a, Supplementary Material online). The origination rate did not show the “J-shape,” but this is due to the removal of genes forming singletons in this study. The “J-shape” pattern can be interpreted in two ways: (i) recent acceleration of evolutionary rates or (ii) lack of data for extinct viral species. The recent acceleration will lead to the evolutionary scenario where most lineages of the nucleocytoviruses become “giant” in the recent period (i.e. RED close to 1). This interpretation is, however, unlikely due to the difficulty in explaining the sudden, recent, and concomitant evolutionary paradigm shift for all nucleocytovirus lineages. It is more plausible that the real evolutionary rates in deeper branches were higher than estimated in this study and comparable to those in the recent past. Consequently, extensive gene gain and loss at the level as inferred for the recent past could have actually occurred since the early stages of the evolution of nucleocytoviruses.

## Conclusion

In summary, we systematically quantified the contributions of different evolutionary mechanisms that shape the gene repertoires of nucleocytoviruses and revealed the previously unrecognized impact of vHGT. We found that vHGT contributes to gene gain at a comparable level to gene duplication and origination. vHGT connects the evolutionary paths of different viruses, allowing for the transmission of genes already adapted to the replication cycle of the donor viruses, such as host range-related genes and arms race-related genes. This means that the large genetic pool of nucleocytoviruses is evolutionarily maintained in the web of gene flow reinforced by vHGT. Furthermore, individual viruses would benefit from being a member of this genomic communication web. Notably, such vHGT can even occur between taxonomically unrelated viruses. This was recently demonstrated by the discovery of a massive vHGT between nucleocytoviruses (the realm *Varidnaviria*) and mirusviruses (the realm *Duplodnaviria*) (Gaïa et al. 2023). We also showed that the vHGT highways are also used by selfish genetic elements to colonize different viruses, as illustrated by the case of insect-infecting viruses. Future studies could provide a more accurate quantification of the gene repertoire evolution of viruses by appropriately including environmental viral genomic data. The mechanism and frequency of gene transfer during coinfection also require further experimental exploration.

## Materials and Methods

### Collection of Reference and Complete *Nucleocytoviricota* Genomes

The reference genomes of viruses in the phylum *Nucleocytoviricota* were collected from the National Center for Biotechnology Information RefSeq databases by searching for the taxonomy *Nucleocytoviricota* or collected from published nucleocytoviruses isolation paper (genomic data are available in www.genome.jp/ftp/db/community/vHGT/vHGT_data/). Our curated dataset includes 195 viruses that cover six proposed viral orders: *Algavirales*, *Asfuvirales*, *Chitovirales*, *Imitervirales*, *Pandoravirales*, and *Pimascovirales*.

### Reconstruction of the Robust Viral Tree

Each virus in the dataset was subject to de novo protein sequence prediction to unify the gene prediction quality using Prodigal/2.6.3 (Hyatt et al. 2010) with the -a parameter to predict all potential genes. From these predicted proteins, we identified and aligned seven suggested marker genes (i.e. poxvirus late transcription factor VLTF3, packaging ATPase, DNA topoisomerase II, transcription initiation factor IIB, DNA polymerase family B, RNA polymerase large subunit, and DEAD/SNF2-like helicase) using the tool ncldv_markersearch (Moniruzzaman et al. 2020) with the -c parameter to produce a multiple sequence alignment file by Clustal Omega/1.2.4 (Sievers and Higgins 2018). To limit the influence of noninformative sites, alignment columns containing more than 90% gaps were trimmed using TrimAI/1.4.1 (Capella-Gutiérrez et al. 2009) with the -gt 0.1 parameter.

To minimize the influence of long-branch attraction effects, we employed the posterior mean site frequency model, which necessitates a guide tree as input. We constructed this guide tree using IQ-TREE/2.2.0 (Minh et al. 2020), selecting the best-fit model by ModelFinder (-m MFP; Kalyaanamoorthy et al. 2017). The final phylogenetic tree was reconstructed under the parameters (-ft < guide tree > -m Q.pfam + F + I + I + R8 + C60 -B 1000 -alrt 1000). For this, Q.pfam + F + I + I + R8 is the optimal model selected in the guide tree and the C60 matrix is used to implement the PMSF model (Wang et al. 2018). -B and -alrt stand for the ultrafast bootstrap [UFboot, (Hoang et al. 2018)] value and SH-aLRT test (Anisimova et al. 2011), respectively. The criterion that we used for the branch support was UFboot > 95% or SH-aLRT > 80% according to IQ-tree documentation. Both iTOL v6 (Letunic and Bork 2021) and Anvi’o v7.1 (Eren et al. 2021) were used to visualize the viral tree.

### Reconstruction of Gene Trees

The predicted genes within the 195 viral genomes were grouped into OGs using OrthoFinder/2.5.4 (Emms and Kelly 2019) with -og parameters, yielding 8,876 OGs excluding singletons. Each OG was aligned using MAFFT/7.505, employing the E-INS-I model (Katoh and Standley 2013) (–maxiterate 1000 –genafpair). Alignment columns consisting of more than 90% gaps were trimmed using TrimAI/1.4.1 to reduce the influence of noninformative sites. These alignments were then subjected to maximum likelihood gene tree reconstruction, leveraging the model that minimizes the Bayesian information criterion via IQ-TREE/2.2.0 (-m MFP). During the tree reconstruction process, we documented 1,000 bootstrap trees (-wbtl) to be used for subsequent tree reconciliation analysis.

Of these 8,876 OGs, 5,285 OGs containing four or more genes were compared with the viral tree for evolutionary reconciliation by ALE. Among them, the tree reconciliation was successful for 4,782 OGs (see details below).

We identified viral-specific OGs among these 4,782 OGs through a sensitive homology search using Diamond/2.0.15 (Buchfink et al. 2021) (–very-sensitive). An OG was classified as viral-specific if none of its members matched any entry in the database, which comprised both Kyoto Encyclopedia of Genes and Genomes cellular organisms (Kanehisa et al. 2023) and metagenome-assembled genomes from marine planktonic eukaryotes, derived from the *Tara* Oceans project (Delmont et al. 2022), with an *E*-value threshold of 10^−3^. Consequently, we identified 3,340 of the 4,782 OGs as being viral-specific.

### Gene Tree and Viral Tree Reconciliation

The previously acquired viral tree and set of gene trees (the bootstrap trees for each OG rather than the consensus tree) were then subjected to the tree reconciliation tool ALE/1.0 (Szöllősi et al. 2013) with the default parameters.

ALE is a probabilistic tool used to explore both gene-level and species-level events within a phylogenetic context. This method reconciles a collection of gene trees, such as a set of bootstrap trees from a gene family, with a predetermined species tree. Ancestral events, such as originations, duplications, transfers, and losses, can thereby be inferred. Furthermore, it enables gene counting at every node of the species tree. ALE accounts for uncertainties of individual gene trees, which are often poorly resolved because of the low information carried in these genes. This approach calculates conditional clade probabilities, representing the weight of a certain gene tree topology from bootstrap samples (ALEobserve), and samples 100 reconciliations in the whole tree reconciliation space to avoid solely explaining the evolutionary scenario by the maximum likelihood reconciliation (ALEml_undated) (Szöllősi et al. 2015). We directly used the raw results, applying integer rounding only after summing the values. For example, we rounded the total number of vHGT events to the nearest integer after calculating the sum. The direct use of raw data takes into account the uncertainty during the inference of evolutionary events.

For some OGs (*n* = 499), ALE reconciliations were unsuccessful. These fell into two cases: (i) they lacked sufficient informative genes within the gene family to conduct a bootstrap test or (ii) after trimming, they contained too many identical sequences, resulting in a nonbifurcated gene tree that hindered subsequent reconciliation.

### Validation of the Contribution of vHGT Using Ranger-DTL

To consolidate the contribution of vHGT in viral evolution, we utilized a different tree reconciliation tool: Ranger-DTL. Unlike ALE, which is based on a probabilistic model, Ranger-DTL operates on a parsimony-based model. This model necessitates the assignment of specific “costs” to various evolutionary events, including gene duplication, transfer, and loss. These costs represent the relative difficulty of each event occurring. The choice of these costs is critical because it significantly influences the results by potentially introducing bias. To mitigate this, we compared gene trees with species trees using various cost settings centering around a set of cost (D/T/L = 3:3:1), which was suggested from the ALE inference result. To reduce bias, we conducted 100 reconciliation trials for each set of costs for the reconciliation between a given gene family and the viral tree. From these trials, we recorded the median value of each gene family and summed these values together to represent the overall events that happened in the set.

### Validation of the Contribution of vHGT by Considering the Presence of Introns

Some viral lineages of *Nucleocytoviricota* contain introns. The existence of introns can lead to the fragmentation of the genes in our gene calling process. Therefore, we performed an additional analysis of the gene families that potentially contain introns. For each OG, the longest sequence was selected as the reference and then the remaining sequences were compared with the reference sequence using Diamond (*E*-value < 1 × 10^−3^). We then considered the relationship between different hits for the predicted genes from each virus. If the neighboring genes matched to the reference sequences, then they were merged by concatenating their sequences. Two genes in a viral genome were considered neighboring genes if there were only <4 genes between them. We set the maximum number of predicted genes that are merged in this process to be four. Using this procedure, we identified 572 gene families as potentially containing genes with introns. The ALE evolutionary reconstructions were performed for the updated gene families.

### Calculation of Tree Certainty for Gene Families

We conducted the tree certainty analysis and computed TCA (Kobert et al. 2016) for all gene families analyzed in this study by using RAxML/8.2.13.AVX2.PTHREADS (-f i -L MRE -z bootstrap_trees -m GTRCAT).

### Calculation of vHGT Frequencies

We normalized the frequency of vHGT using the sizes of two specific lineages. vHGT occurring between two lineages can be listed in a matrix, in which the diagonal values represent the intra-lineage vHGTs. The normalization process converted the raw counts of vHGT events into frequencies by accounting for the size of each lineage involved. This calculation is represented as follows: *F*_vHGT_ = No_vHGT_/(*N*_lineage1_ × *N*_lineage2_), where No_vHGT_ is the number of inferred vHGT events between two lineages, *N*_lineage1_ is the number of members in the first lineage, and *N*_lineage2_ is the number of members in the second lineage. After calculating these frequencies, we scaled them so that the maximum value was set to 1 and the minimum value was set to 0.

### Functional Analysis

For each gene family, the best GVOG hit (Aylward et al. 2021) was identified for each gene family member using Diamond (*E*-value < 1 × 10^−3^). Then, the GVOG with the largest number of best hits was assigned to the gene family. EggNOG classifications for individual families were derived from the original table of GVOG (Aylward et al. 2021).

### Analysis of the Evolutionary Event Rate

RED (Parks et al. 2018), which forms the basis for analyzing the dynamics of the evolutionary event rate, was computed using the “get_reds” function of the “castor” R package (Louca and Doebeli 2018). The normalized evolutionary rate was then determined by taking the natural logarithm of the number of events (plus one) at each node divided by the corresponding branch length leading to that node. We performed the Mann–Whitney *U* test using the R package “ggsignif” (Ahlmann-Eltze and Patil 2021) to examine the rate differences of the various evolutionary events. The ANI was calculated by fastani/1.33 (Jain et al. 2018).

## Supplementary Material

msae161_Supplementary_Data

## Data Availability

Data supporting the findings of this study are available within the article and its supplemental files as well as at the GenomeNet FTP: https://www.genome.jp/ftp/db/community/vHGT/vHGT_data/.
