# Supplementary material for: Gene Transfer Among Viruses Substantially Contributes to Gene Gain of Giant Viruses
Source: Mol Biol Evol. 2024 Aug 2;41(8):msae161. doi: 10.1093/molbev/msae161 (PMC11334073; doi:10.1093/molbev/msae161)
Supplement: msae161_Supplementary_Data [file msae161_supplementary_data.zip › Wu et al Supplementary Tables and Figures_240705.pdf]

Gene transfer among viruses substantially contributes to gene gain of giant viruses

Junyi Wu[1], Lingjie Meng[1], Morgan Gaïa[2,3], Hiroyuki Hikida[1], Yusuke Okazaki[1], Hisashi Endo[1], Hiroyuki Ogata[1,*]

Affiliations:

1. Bioinformatics Center, Institute for Chemical Research, Kyoto University, Gokasho, Uji, 611-0011, Japan

2. Génomique Métabolique, Genoscope, Institut François Jacob, CEA, CNRS, Univ Evry, Université Paris-Saclay, F-91057 Evry, France

3. Research Federation for the study of Global Ocean systems ecology and evolution, FR2022/Tara GOsee, F-75016 Paris, France

***Corresponding author:**

Hiroyuki Ogata (Email: ogata@kuicr.kyoto-u.ac.jp, Phone: +81-774-38-3270)

Supplementary Materials

- **Table S1–S3**
- **Figure S1–S10**
- **Supplementary Data (in a separate Excel file).**

Table S1. Examples of the number of evolutionary events summarized from root to individual virus.

Viruses	Order	Genes for Reconciliation	Total Gains	Total Losses	Log(Gains / Losses)
Paramecium bursaria Chlorella virus NY2A	Algavirales	346	545	204	0.9826658
Ostreococcus tauri virus OtV5*	Algavirales	202	424	243	0.5566720
Kaumoebavirus strain KLCC10	Asfuvirales	224	273	28	2.2772673
Abalone asfarvirus*	Asfuvirales	55	128	69	0.6179238
Melanoplus sanguinipes entomopoxvirus	Chitovirales	187	239	38	1.8388774
Cotia virus SPAn232*	Chitovirales	157	365	199	0.6065925
Tupanvirus soda lake	Imitervirales	770	1120	263	1.4489299
Namao virus*	Imitervirales	110	354	270	0.2708750
Emiliania huxleyi virus 202	Pandoravirales	395	563	143	1.3704350
Mollivirus sibericum*	Pandoravirales	165	327	200	0.4916428
Orpheovirus IHUMI-LCC2	Pimascovirales	557	693	140	1.5993876
Dikerogammarus haemobaphes virus 1	Pimascovirales	11	178	201	-0.1215214
Lymphocystis disease virus 1	Pimascovirales	56	239	218	0.0919685

This table show selected examples of viruses from each viral order. We selected viruses with the highest, lowest, and closest to zero values for the log-transformed ratio of gene gain events to gene loss events. A positive log-transformed value indicates a tendency of gene gains over losses, while a negative value indicates a higher frequency of gene losses over gains. A value of zero signifies an equivalent rate of gene gain and loss during evolution. The viruses with an asterisk (*) showed the lowest value and the value closest to zero within the viral order.

Table S2. Evolutionary events inferred by parsimony-based method, Ranger-DTL, under different sets of cost parameters (D: duplication, T: transfer, L: loss).

Cost	Duplications	Transfers	Originations	D : T : O	Gains	Losses	G/L	Root	*Pokkeviricetes*	*Megaviricetes*
DTL131	6739	16359	4782	24%: 59%: 17%	27880	14334	1.94	0	1	1
DTL331	2968	20077	4782	11%: 72%: 17%	27827	9955	2.8	0	1	0
DTL531	2494	20631	4782	9%: 74%: 17%	27906	9823	2.84	0	4	0
DTL731	2335	21054	4782	8%: 75%: 17%	28171	9723	2.9	0	1	0
DTL931	2329	21077	4782	8%: 75%: 17%	28188	9763	2.89	0	0	0
DTL311	2326	25658	4782	7%: 78%: 15%	32766	2809	11.66	0	0	0
DTL331	2968	20077	4782	11%: 72%: 17%	27827	9955	2.8	0	1	0
DTL351	6641	15615	4782	25%: 58%: 18%	27038	17172	1.57	2	1	5
DTL371	14367	7666	4782	54%: 29%: 18%	26815	40069	0.67	17	1	21
DTL391	16807	5240	4782	63%: 20%: 18%	26829	51778	0.52	34	3	56
DTL331	2968	20077	4782	11%: 72%: 17%	27827	9955	2.8	0	1	0
DTL333	2867	25029	4782	9%: 77%: 15%	32678	2747	11.9	0	0	0
DTL335	2869	26723	4782	8%: 78%: 14%	34374	1235	27.83	0	0	0
DTL337	2937	28477	4782	8%: 79%: 13%	36196	328	110.52	0	0	0
DTL339	2895	29131	4782	8%: 79%: 13%	36808	102	360.86	0	0	0

Columns marked with “Root”, “*Pokkeviricetes*”, and “*Megaviricetes*” represent the inferred number of genes in the ancestral genomes of these clades.

Table S3. Number of evolutionary events inferred for gene families after alleviating the influence of the presence of potential introns.

Order	Duplications	Transfers	Originations	Gains	Losses
Algavirales	96	136	22	254	292
Asfuvirales	138	164	32	334	285
Chitovirales	157	232	65	454	477
Imitervirales	832	846	242	1920	2014
Pandoravirales	218	139	116	473	452
Pimascovirales	364	651	95	1110	1293

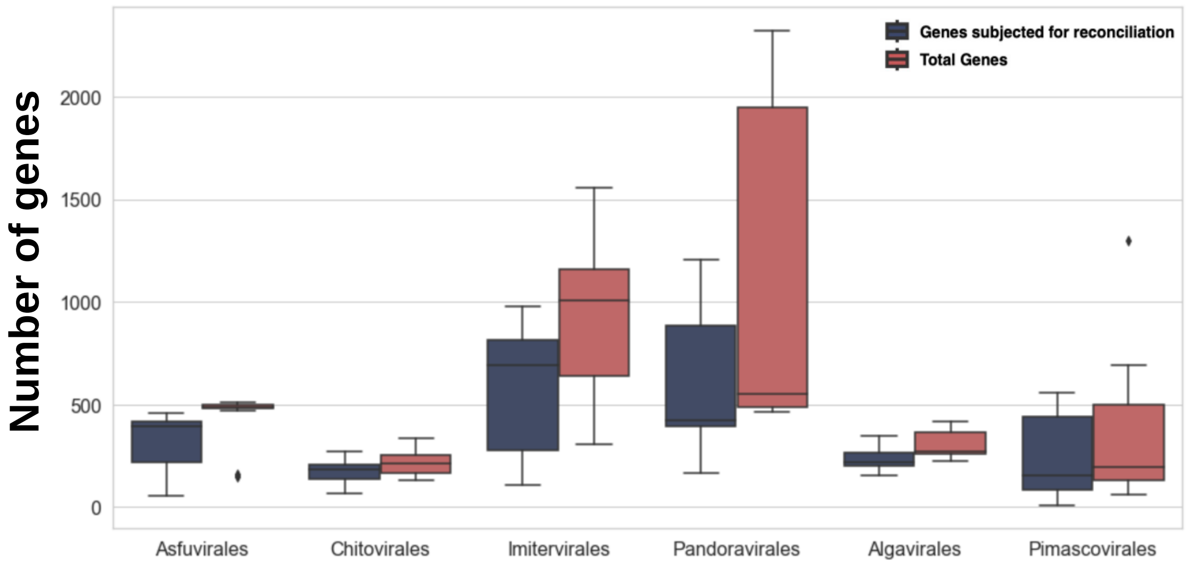

Figure S1. Total number of genes and the number of genes included in the tree reconciliation analysis for individual viral genomes.

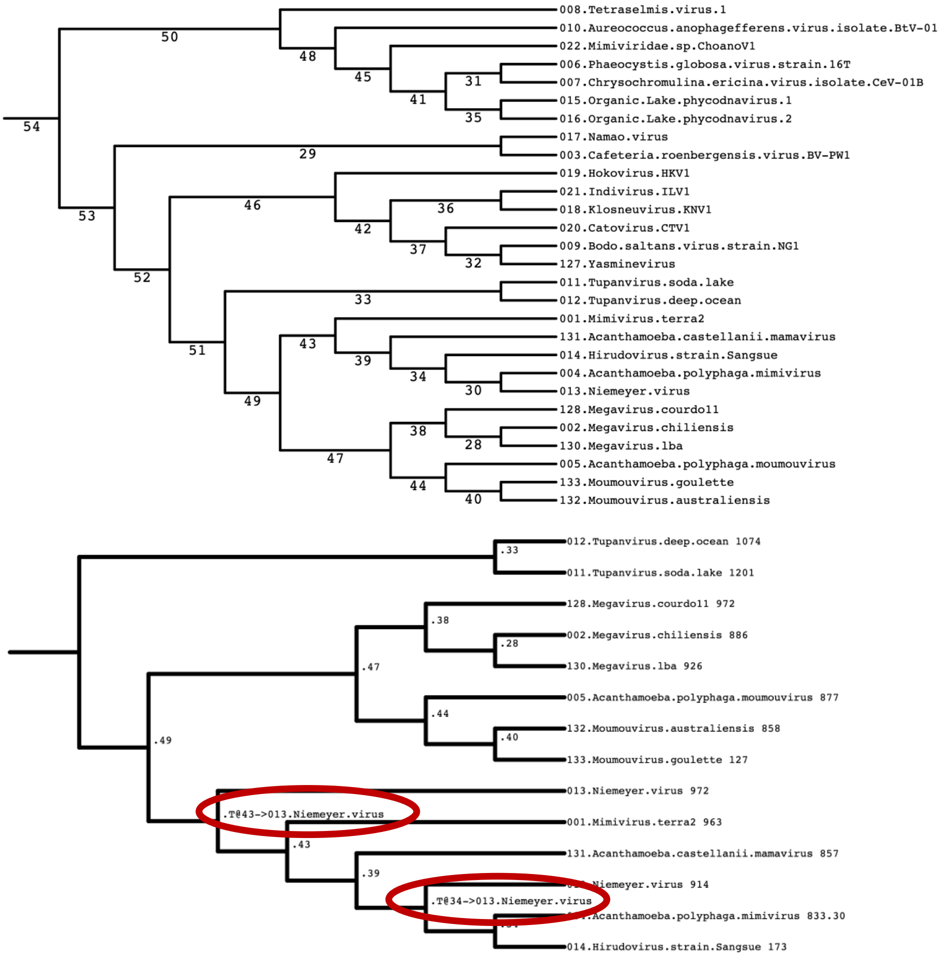

Figure S2. Example of tree reconciliation.

We selected the tree reconciliation result for OG0001042 to visualize inferred vHGT events. Two vHGT events were inferred to explain the existence of two copies of OG0001042 in Niemeyer virus. The upper tree is the subtree of the viral tree for the *Imitervirales* order. The numbers on the branches represent the internal node IDs, the numbers preceding the virus names represent the lief IDs. The lower tree is one of the sampled reconciliation gene tree topologies with annotated events consistent with the final results (i.e., two vHGT events). In this gene tree, the texts beside the internal nodes represent evolutionary events. For example, ".33" indicates that the evolution of this gene in two tupanviruses followed their speciation event (as indicated with "33" in the upper viral tree). The text ".T@43 -> 013.Niemeyer.virus" indicates that there was a vHGT from internal node "43" to Niemeyer virus (leaf "013" in the viral tree). In this case, it is plausible that an unsampled virus branching out from near node "43" was the doner for this vHGT. This figure is provided to illustrate how the concept of tree reconciliation works. However, the tree reconciliation in this figure is one of 100 ALE inferences for this gene family, representing one of the many possible scenarios. Therefore, it should be noted that specific evolutionary events (such as "T@34->013") inferred in this reconciliation may not be a majority or consensus of 100 inferences.

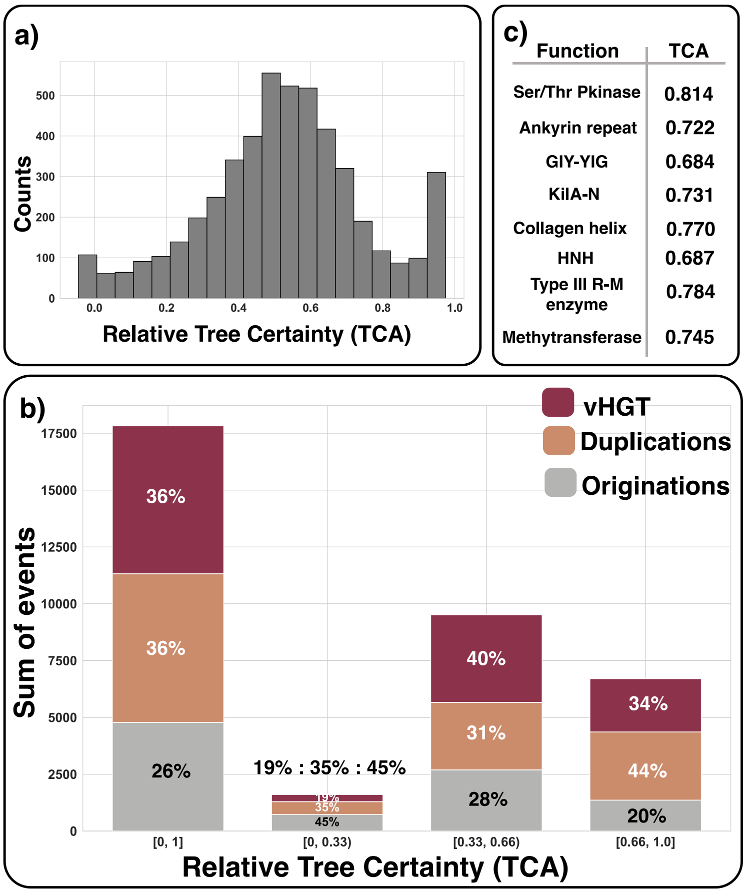

Figure S3. Effects of gene tree stability on the contribution of different gene gain mechanisms

a) The histogram represents the distribution of TCA for all OGs that used in this study. b) The bar plot shows the contribution of different gene gain mechanisms in different range of TCA. c) TCA values for examples same as in the Figure 5.

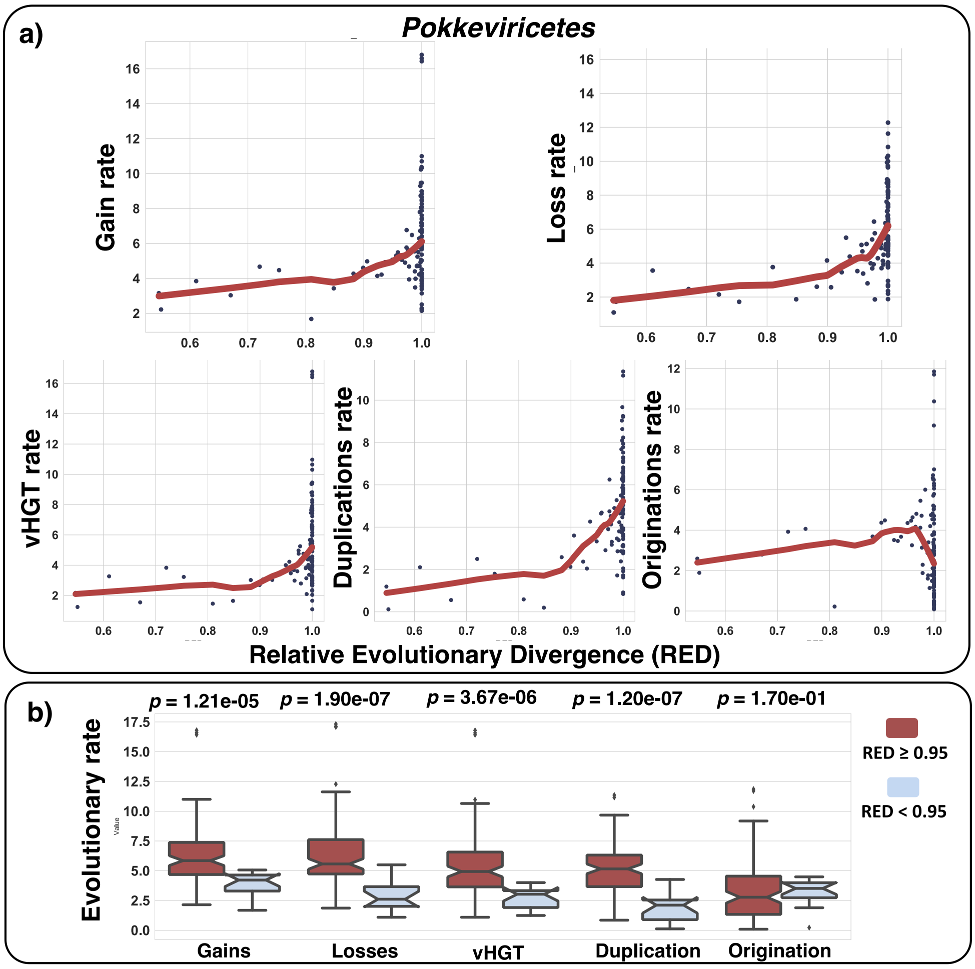

Figure S4. Rates of different evolutionary events along the divergence of *Pokkeviricetes*

a) The evolutionary rates for different evolutionary events are plotted against the divergence measured by RED. The red line represents the local regression with LOESS (with parameter 'frac'=0.9). (b) Boxplot provides a comparison of evolutionary rates between recent (RED ≥ 0.95) and earlier periods (RED < 0.95). *P*-values calculated using the Mann-Whitney U test are shown above the graph.

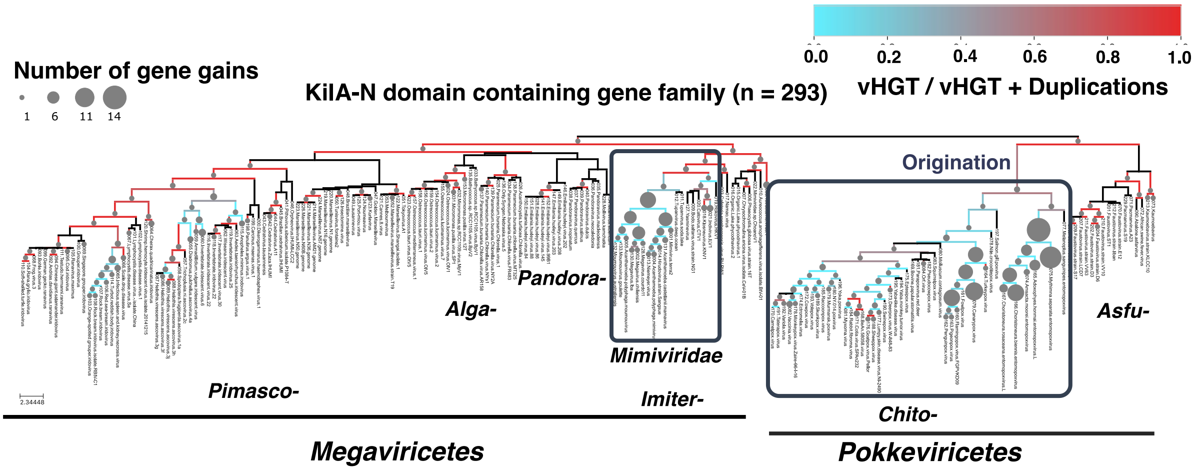

Figure S5. The vHGT propensity against gene duplication in KilA-domain containing gene family. For Figure S5 to S10, the level of vHGT propensity against gene duplication was represented by color from blue (0.0) to red (1.0). The size of the circles at nodes represents the number of gene gains. Lineages with clear gene duplication after vHGT are highlighted.

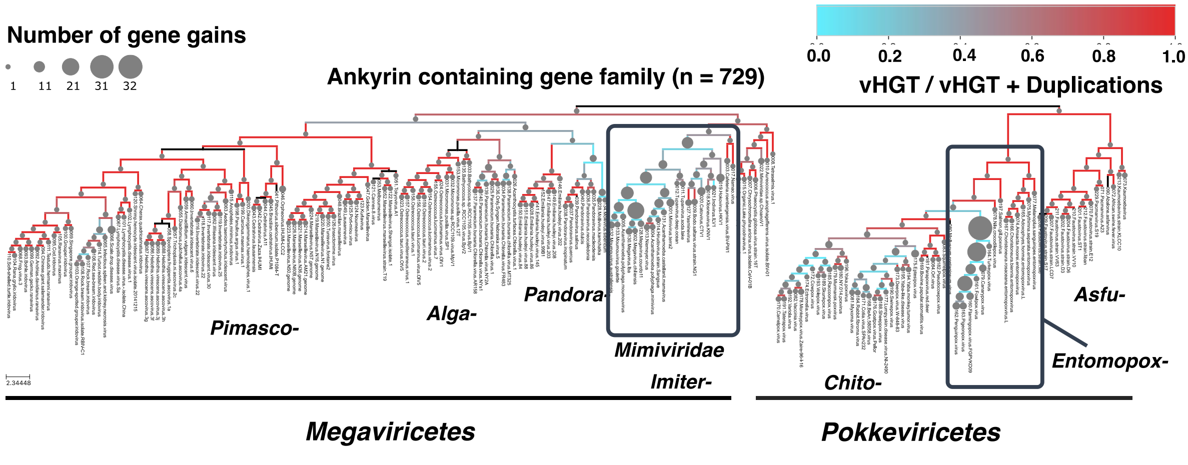

Figure S6. The vHGT propensity against gene duplication in Ankyrin-domain containing gene family.

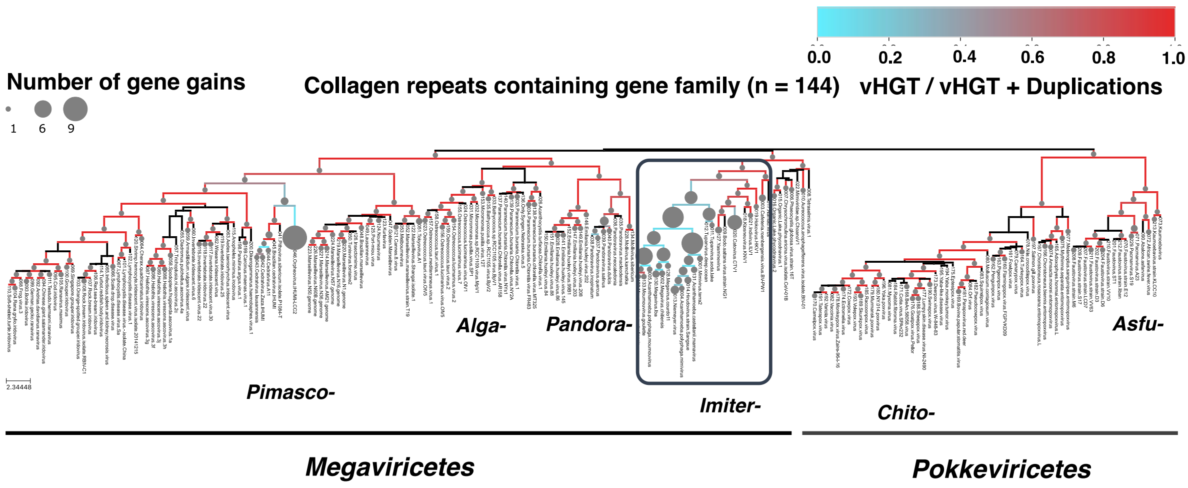

Figure S7. The vHGT propensity against gene duplication in collagen repeats domain containing gene family.

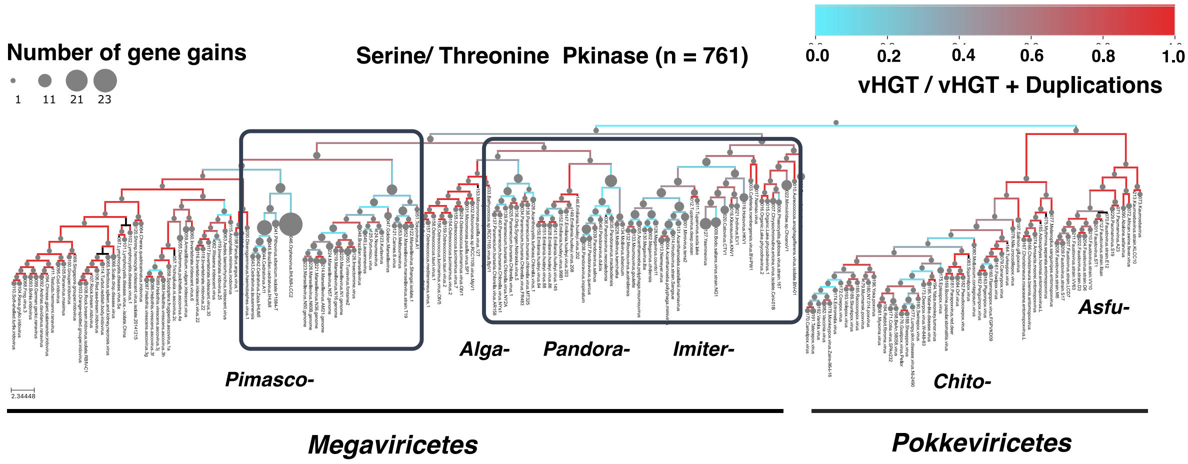

Figure S8. The vHGT propensity against gene duplication in Serine/Threonine protein kinase gene family.

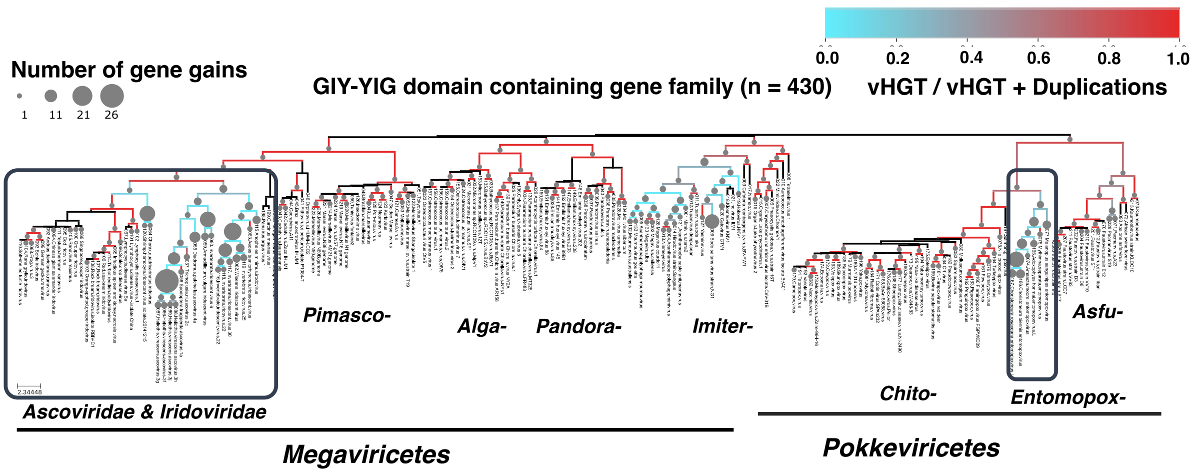

Figure S9. The vHGT propensity against gene duplication in GIY-YIG domain containing gene family.

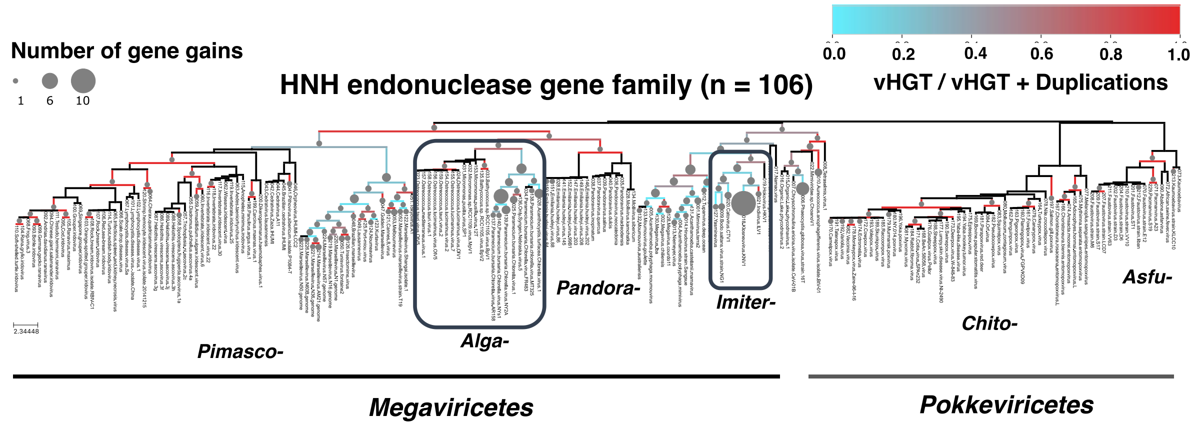

Figure S10. The vHGT propensity against gene duplication in HNH endonuclease gene family.
